# The associations between age, familial occurrence of fibromyalgia, and symptom severity in fibromyalgia: a cross-sectional study from a Finnish health center

**DOI:** 10.1186/s41927-026-00651-x

**Published:** 2026-05-08

**Authors:** Gretel Raudasoja, Aleksi Varinen

**Affiliations:** 1 Faculty of Medicine and Health Technology, Tampere University, Tampere, Finland; 2 Linnainmaa Health Center, Wellbeing Services County of Pirkanmaa, Tampere, Finland; 3 Department of General Practice, Faculty of Medicine and Health Technology, Tampere University, Tampere, Finland; 4 Pain Clinic, Kanta-Häme Central Hospital, Hämeenlinna, Finland

**Keywords:** Fibromyalgia, Symptoms-severity, Aging, Heredity

## Abstract

**Background:**

Fibromyalgia is a functional syndrome characterized by musculoskeletal pain and a variety of associated symptoms. Previous research has shown that close relatives are at a higher risk of developing the syndrome compared to the general population. Previous findings also suggest that symptoms tend to decrease with age. Our primary objective is to examine whether having a close relative with fibromyalgia is associated with greater symptom severity among patients in primary care. In addition, we assess the relationship between age and symptom severity.

**Methods:**

The study is based on a cross-sectional design. The data were collected at the Nokia Health Centre, Finland, in 2016. Patients meeting the ACR 2010 criteria were included in this study (n = 91). We used three validated questionnaires to assess disease severity (PSD, FIQ and EQ-VAS) and patient-reported information on fibromyalgia in a close relative.

**Results:**

The independent-samples t-test was used to examine the association. Participants were divided into four age groups, and differences in symptom severity between age groups were assessed using one-way analysis of variance (ANOVA). There were no statistically significant differences between family history and symptom severity, nor age groups and symptom severity. Furthermore, there was no statistically significant linear association between age and symptom severity, nor between symptom severity and family history of fibromyalgia. These findings remained unchanged after adjusting for family history. However, given the lack of statistical significance and our small sample size, these observations should be interpreted cautiously.

**Conclusions:**

Symptom severity and functional limitations appeared broadly similar across age groups in our sample, which may suggest that increasing age is not necessarily associated with substantial symptom relief. However, these findings should be interpreted with caution given the cross-sectional design and small sample size.

## Introduction

Fibromyalgia is a functional pain disorder defined by chronic widespread pain (CWP) as its core clinical feature. In addition to CWP, which is linked to central sensitization, individuals with fibromyalgia frequently report fatigue, disordered sleep, and memory or concentration problems [1]. Depression, anxiety, gastrointestinal disturbances, and dizziness are also typical comorbidities observed in individuals with fibromyalgia [2].

The diagnosis of fibromyalgia is clinical and based on widespread chronic pain (≥ 3 months) and additional symptoms, mainly fatigue or sleep problems. Various diagnostic criteria have been developed to support diagnostic decision‑making and research. The ACR 2010 criteria provide a clinical framework for diagnosis of fibromyalgia, combining the Widespread Pain Index (WPI) with the Symptom Severity Scale (SS), the latter primarily capturing fatigue- and sleep-related symptoms [1].

Fibromyalgia has a prevalence of approximately 2–4% in the general population [3]. Although it was previously considered a psychogenic disorder, more recent research suggests that it is associated with neural disturbances in pain processing, pain regulation, and altered stress response. However, the underlying pathophysiology of fibromyalgia is still somewhat unclear. Known risk factors for fibromyalgia include genetic factors, female sex, sleep disturbances, low physical activity and overweight or obesity [3].

### The genetics of fibromyalgia

Exact genetic factors predisposing individuals to fibromyalgia remain unclear [4]. However, the findings from epidemiological studies support contributions from both genetic predisposition and unique environmental influences to the development of CWP [2]. The heritability of widespread pain alone has been studied in several twin studies, in which its heritability has been estimated to be approximately 50% [5]. In addition, a family study of fibromyalgia indicates that siblings of fibromyalgia patients are 13.6 times more likely to develop the syndrome compared to the general population [6]. Genetic variation within serotonergic, dopaminergic, and catecholaminergic pathways has been proposed to contribute to susceptibility to fibromyalgia. The specific contribution of these polymorphisms to symptom severity remains uncertain, although variants related to pain processing, inflammation, and oxidative stress have been suggested to play a role [4, 7, 8].

According to a large national registry study involving 5.8 million individuals, patients with fibromyalgia also have a significant genetic risk profile for several other diseases, such as functional somatic disorders, pain syndromes, psychiatric disorders, autoimmune disorders, and sleep disorders [9].

There is also evidence of environmental influences on gene expression [2]. Therefore, a patient’s lifetime experiences, such as long-term stress, may have an impact on the onset of diseases. For example, peer bullying in childhood may be linked to fibromyalgia later in life [10]. In addition, an association between higher FIQ score and family stress factors, such as illness, retirement, death and birth was found in a study from the United States [11]. However, it seems that certain shared childhood environmental factors such as parenting have no association with susceptibility to the syndrome [2].

### Factors associated with symptom severity

Several factors have been associated with fibromyalgia disease severity. Low socioeconomic status has been shown to affect the severity of symptoms in patients with fibromyalgia [12]. Lower educational attainment has also been found to significantly influence symptom severity, quality of life, and functional capacity compared with patients with higher levels of education.

The impact of obesity has also been a subject of interest over the past couple of decades, and there is increasing evidence of an association between pain and overweight or obesity [13]. In addition, a Finnish study found that fibromyalgia patients with significantly elevated hsCRP (high-sensitivity C-reactive protein) levels also had more severe symptoms measured by higher FIQ scores and greater pain [14]. However, these patients were also overweight and had lower physical activity levels, which likely explained the association through systemic inflammation.

Furthermore, Buskila et al. analyzed fibromyalgia symptoms in offspring of mothers with fibromyalgia [15]. There was no significant association between offspring with or without fibromyalgia and psychological factors (depression and anxiety), global well-being, or quality of life scores. However, the offsprings with fibromyalgia had significantly more pain, fatigue, and morning stiffness compared with offspring without fibromyalgia.

## Fibromyalgia and age

The onset of fibromyalgia can occur at any age, even in childhood [16]. According to previous research, patients with fibromyalgia who have a hereditary predisposition are diagnosed at a younger age, whereas those diagnosed at an older age may have had symptoms of nociceptive pain possibly facilitating the process of pain sensitization [17].

Previous research suggests that fibromyalgia symptom profile improves with aging [18–20]. These studies show that, in older patients, the disease had less impact on both physical and social functioning compared with younger patients. On the other hand, an Italian study showed a more steady‑state disease course across age groups [21]. This study showed a bimodal pattern in revised FIQ scores, with individuals over 70 years and 50–60 years having more severe symptoms than individuals aged 60–70. However, the differences were not statistically significant. In many Western countries, individuals typically retire at around 65 years of age, and retirement has been associated with health improvements—such as increased physical activity and longer sleep duration—which could partly explain these findings [22, 23].

In conclusion, previous research has identified many factors underlying the genetic susceptibility to fibromyalgia, as well as individual factors influencing disease severity. Nevertheless, the overall picture remains unclear, and further studies are needed.

This study uses data collected from patients with fibromyalgia at the Nokia Health Centre in Finland. Our objective is to assess whether symptom severity among primary care patients diagnosed with fibromyalgia is related to their report of having a close relative with the condition. To the best of the author’s knowledge, there are no previous studies on this topic. In addition, we aim to examine the relationship between age and symptom severity.

## Materials and methods

The study is based on a cross-sectional design. The dataset was created in 2016 by identifying all patients at the Nokia primary healthcare centre who met the eligibility criteria up to that point [24, 25]. At the time, the city of Nokia had 33,210 inhabitants and 19 primary healthcare physicians working at the city health centre. Inclusion criteria were an ICD-10 code M79.8 or a fibromyalgia diagnosis and other ICD-10 codes (M79.0, M25.5, R52.9, and M79.1) that included the word “fibromyalgia” or “fibrositis” in patients’ medical record text. Participants were excluded if they were younger than 18 years or lacked sufficient proficiency in reading, writing, or speaking Finnish to complete the study procedures. Additional exclusion criteria comprised severe cognitive impairment (e.g., Alzheimer’s disease), psychotic-level psychiatric disorders, and unstable medical conditions (such as metastatic cancer). Individuals who were pregnant or breastfeeding at the time of recruitment were also excluded.

A systematic retrieval of electronic patient records was conducted to identify all available fibromyalgia cases, resulting in 208 patients [24, 25]. These patients were sent an invitation letter with questionnaires, and 103 responded. There were no statistically significant differences between respondents who consented to participate and individuals who did not respond to the study letter with respect to sex, age, number of regular medications, number of physician visits during the preceding 12 months, or the number of chronic conditions [24]. All respondents (*n* = 103) were scheduled for single study appointment at general practice, during which they completed the ACR 2010 form (The American College of Rheumatology 2010 diagnostic criteria for fibromyalgia) [1].

All data for this study were derived from the study visit. Disease severity was assessed using three validated questionnaires. Polysymptomatic Distress Scale (PSD) is a direct composite score derived from the ACR2010 criteria and consists of the Widespread Pain Index (WPI) and the Symptom Severity Scale (SS) [26]. The diagnostic criteria for fibromyalgia require a score of at least 12 points on PSD scale (maximum 31) with higher scores indicating greater symptom burden. The WPI and the SS form were completed during the doctor’s appointment in general practice [24, 25].

The Fibromyalgia Impact Questionnaire (FIQ) is a validated method for measuring functional capacity [27]. The FIQ score ranges from 0 to 100, with higher values indicating greater interference of symptoms with daily functioning. The FIQ form was sent to study population with the invitation letter [24, 25].

The EuroQol Visual Analogue Scale (EQ-VAS) assesses a patient’s health status on a scale of 0 (the worst imaginable health) to 100 (the best imaginable health) [28]. The EQ-VAS is part of the EQ-5D-5 L form, which was sent to study population with the invitation letter [24, 25].

Patient‑reported information about fibromyalgia in a close relative was collected during the study visit using the question: “*Has any of your close relatives ever been diagnosed with fibromyalgia?*”. Participants who answered “yes” were classified as having a family history of fibromyalgia, whereas those who answered “no” or reported being unaware of a diagnosis in a close relative were classified as having no family history of fibromyalgia.

## Statistical methods

Frequencies and percentages were reported for categorical variables (gender and family history of fibromyalgia), and means and standard deviations were reported for continuous variables (age and questionnaire scores).

Crosstabulations were analysed with the chi-square and Fisher’s exact test to assess gender differences in family history of fibromyalgia. All continuous variables followed a normal distribution. Normality was initially evaluated by visual inspection. This was followed by a Kolmogorov–Smirnov test, which provided no statistically significant evidence against the assumption of normality.

Independent-samples t-test was performed to analyse whether family history of fibromyalgia was associated with symptom severity or age. Patients were divided into four age groups: <45 years, 45–54 years, 55–64 years, and ≥ 65 years. Differences in symptom severity between age groups were assessed using one-way analysis of variance (ANOVA).

We also conducted additional analyses examining the relationships among age, symptom severity, and family history of fibromyalgia using Pearson’s correlation coefficient and partial correlation.

In all statistical tests, a p-value below 0.05 was considered statistically significant. The data were analysed using IBM SPSS Statistics version 29.

This research was carried out in accordance with the Declaration of Helsinki. Ethical approval was obtained from the Regional Ethics Committee of Tampere University Hospital, and informed consent was secured from all participants [1].

## Results

**Fig. 1:**
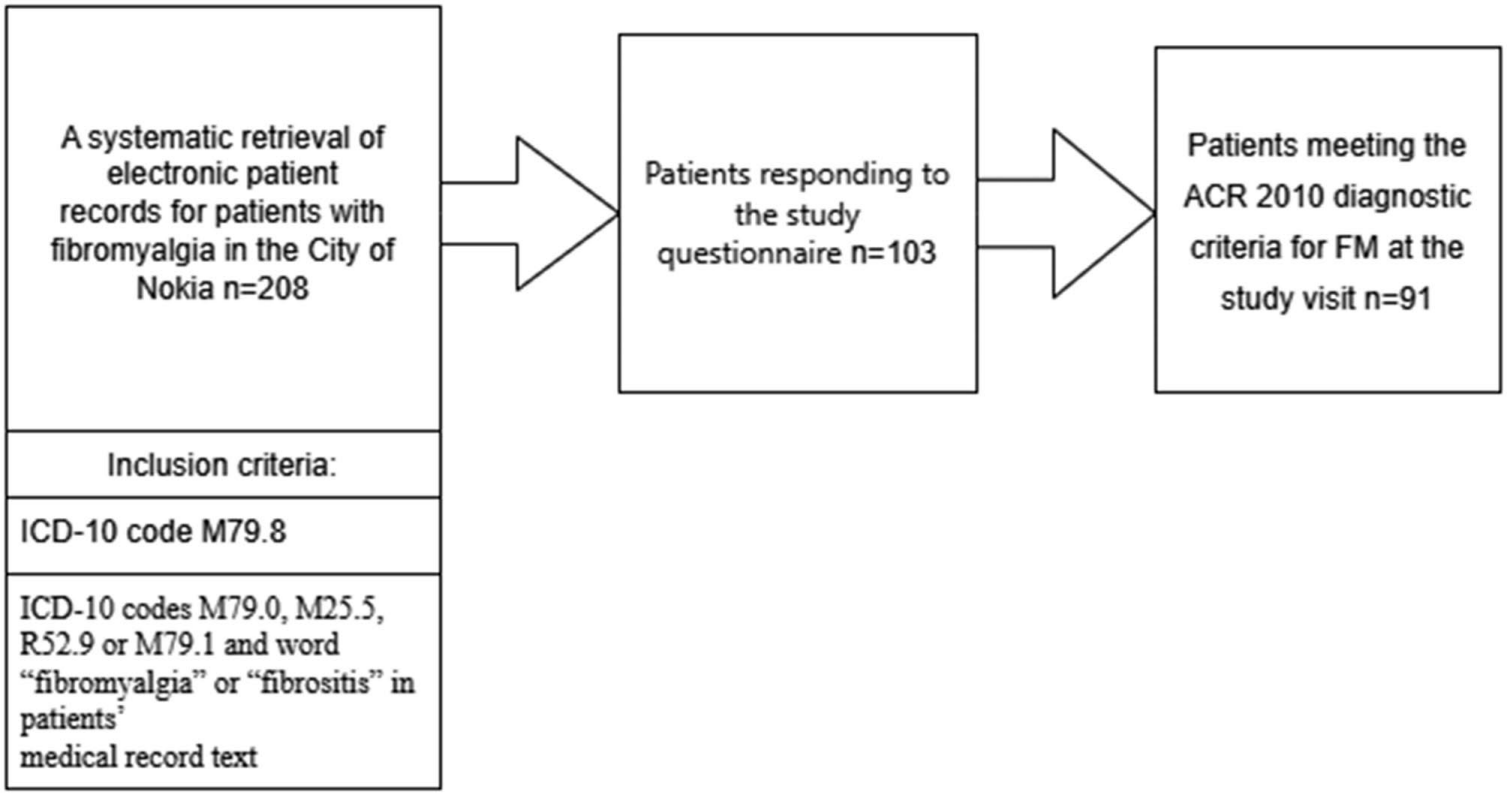
Flowchart of study population

Finally, 91 participants (83 female and 8 male) who met the ACR 2010 fibromyalgia diagnostic criteria were included in this study (Fig. 1). Their ages ranged from 18 to 85 years. Some forms were not completed properly; the number of individuals who completed the FIQ and EQ-VAS is smaller [24] (Table 1).

There was no statistically significant difference between family history of fibromyalgia and gender. A slight, non‑significant tendency toward younger age was observed in the family-history group, although the relevance of this finding remains uncertain. There was also no statistically significant difference between the family history groups and the three questionnaire scores (Table 1).

**Table 1:** Characteristics of participants and the association between family history for FM (fibromyalgia) and symptom severity

		All	No family history	Family history for FM	*p*-value
		*N* = 91	*N* = 80	*N* = 11	
Male	n (%)	8 (100)	7 (87,5)	1 (12,5)	
Female	n (%)	83 (100)	73 (88)	10 (12)	0.659
Age	mean (SD)	54.66 (15.36)	55.47 (15.25)	48.73 (15.61)	0.173
PSD*	mean (SD)	21.70 (3.71)	21.64 (3.73)	22.18 (3.68)	0.651
FIQ*	mean (SD)	46.77 (14.67)	46.95 (14.57)	45.58 (15.92)	0.775
EQ-VAS*	mean (SD)	48.84 (19.09)	48.63 (19.55)	50.36 (16.19)	0.779

*Number of completed forms: PSD, n = 91; FIQ, n = 82; EQ-VAS, n = 90

PSD = Polysymptomatic Distress Scale, FIQ = Fibromyalgia Impact Questionnaire, EQ-VAS = The EuroQol Visual Analogue Scale

**Fig. 2:**
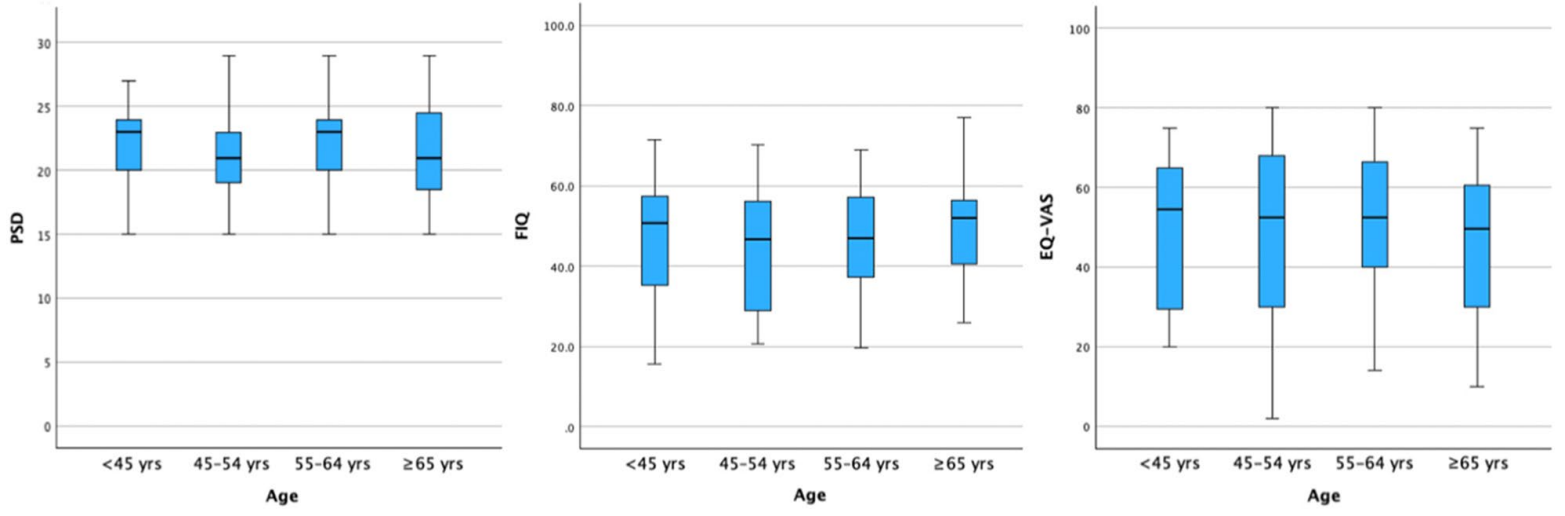
Boxplots of symptom severity across age categories, showing the median (black line), interquartile range (blue area), and minimum and maximum values

When divided into four age groups, ANOVA indicated no statistically significant differences in symptom severity among the groups (Table 2; Fig. 2).

**Table 2:** The association between age group and disease severity (the full unadjusted sample)

		< 45 years	45–54 years	55–64 years	≥ 65 years	*p*-value
PSD	*n*	25	14	24	28	
	mean (SD)	21.80 (3.24)	21.50 (4.07)	22.42 (3.71)	21.11 (4.00)	0.651
FIQ	n	23	14	22	23	
	mean (SD)	46.37 (15.03)	43.21 (15.86)	45.94 (14.48)	50.09 (14.03)	0.568
EQ-VAS	n	24	14	24	28	
	mean (SD)	48.81 (19.50)	48.00 (23.68)	52.54 (17.87)	46.11 (17.76)	0.689

PSD = Polysymptomatic Distress Scale, FIQ = Fibromyalgia Impact Questionnaire, EQ-VAS = The EuroQol Visual Analogue Scale

In our additional analyses, zero‑order (Pearson) correlations indicated no statistically significant associations among age, family history of fibromyalgia, and the symptom severity scales (PSD, FIQ, EQ‑5D‑VAS). Effect sizes were uniformly small (|*r*| ≤ 0.16), and adjustments did not meaningfully alter these relationships.

### PSD

Age was not correlated with PSD (*r* = − 0.091, *p* = 0.391; df = 89) or with family history (*r* = − 0.144, *p* = 0.173; df = 89). PSD also showed no association with family history (*r* = 0.048, *p* = 0.651; df = 89). The partial correlation between age and PSD, adjusting for family history, was similarly small and non-significant (*r* = − 0.085, *p* = 0.425; df = 88).

### FIQ

Age was not correlated with the FIQ score (*r* = 0.099, *p* = 0.378; df = 80) or with family history (*r* = − 0.134, *p* = 0.228; df = 80), and FIQ score was also not associated with family history (*r* = − 0.032, *p* = 0.775; df = 80). Controlling for family history did not materially change the age–FIQ relationship (*r* = 0.095, *p* = 0.397; df = 79).

### EQ-5D-VAS

Age showed no significant correlation with EQ-5D-VAS (*r* = − 0.10, *p* = 0.331) or with family history (*r* = − 0.16, *p* = 0.134). EQ-5D-VAS was likewise not correlated with family history (*r* = 0.03, *p* = 0.779). In a partial correlation controlling for family history, the age–EQ-5D-VAS association remained non-significant (*r* = − 0.10, *p* = 0.350; df = 87).

## Discussion

We did not observe statistically significant differences between individuals with and without a family history of fibromyalgia in terms of symptom severity; however, these findings should be interpreted cautiously, as the small number of participants with a family history may limit the statistical power to detect potential associations. In addition, although the mean age of participants in the family‑history group was 6.7 years lower than in the no‑family‑history group, this difference was not statistically significant and should therefore be considered descriptive rather than indicative of a true underlying effect. Still, the observed age difference may reflect underlying factors related to family history, although any such explanation remains speculative given the lack of statistical significance [17].

Familial occurrence of fibromyalgia did not differ statistically between genders. However, the number of male participants was very low. There were no statistically significant differences between family history and no-family-history group when assessed using the three selected questionnaires. In the PSD, the family‑history group had a mean score 0.54 points higher, whereas in the FIQ, the no‑family‑history group had a mean score 1.4 points higher, indicating greater functional impact. However, these differences were not statistically significant and should therefore be interpreted with caution and considered descriptive rather than indicative of any meaningful underlying effect. Finally, in the EQ‑VAS, the family‑history group had a mean score 1.7 points higher, indicating better perceived health status compared with the no‑family‑history group. However, none of these differences were statistically significant.

In addition, our results provide no evidence of meaningful linear associations between age and the studied outcomes (EQ‑5D‑VAS, PSD, FIQ), nor between these outcomes and family history of fibromyalgia; controlling for family history did not alter these conclusions. However, these findings should be interpreted with caution given the relatively small sample size, which may limit the statistical power to detect potential associations.

Comparing different age groups, the 55–64‑year age group had the most symptomatic disease as measured by the PSD. However, the group means differed by at most 1.3 points. The FIQ means showed a bimodal pattern: the youngest and the oldest age groups had the greatest impact on functioning, whereas the 45–54‑year group had the least impact. This is to some extent in line with earlier research, but the differences observed were small and likely not clinically relevant [21]. The maximum difference in FIQ means across age groups was 6.9 points. Finally, according to the EQ‑VAS, the 55–64‑year age group appeared to have the best health‑related quality of life compared with the other groups. However, none of these differences between age groups were statistically significant. Our results therefore showed no statistically significant differences between age groups, which is consistent with one previous study, whereas another study reported a linear association between age and fibromyalgia symptom severity [21].

Strengths of our study include a fairly comprehensive sample of patients with fibromyalgia from one large health centre in Finland, the inclusion of both female and male participants, and a diagnosis of fibromyalgia confirmed by a general practitioner according to the ACR 2010 diagnostic criteria. Furthermore, we used validated questionnaires to assess the impact of fibromyalgia syndrome. Another strength of our study is the reported proportion of patients with a family history of fibromyalgia, which contributes valuable data to a field where estimates of familial occurrence and heritability remain limited.

There are several weaknesses in our study. The number of participants with a family history of fibromyalgia was quite low, and information on family history was self‑reported. There was also some missing data, as some participants had not completed all questionnaires fully. In addition, the FIQ form includes several work‑related questions; because some participants were retired, they received lower scores on the functional ability section for reasons unrelated to disease severity.

Furthermore, the data were collected from only one health centre, which may limit the generalizability of the results. Another limitation is the lack of covariate adjustment. Although factors such as employment status and environmental exposures may influence the development or severity of fibromyalgia and could bias the results if uncontrolled, the small sample size made covariate inclusion statistically inappropriate. Following consultation with a statistician, most analyses were therefore conducted without covariate adjustment, which may reduce the interpretability of the findings.

Finally, an additional limitation is that family history of fibromyalgia was reported without distinguishing first‑degree from more distant relatives. This may introduce misclassification bias, including recall bias or limited awareness of relatives’ diagnoses.

Regarding age, we decided to divide the study population into four age subcategories (< 45 years, 45–54 years, 55–64 years, and ≥ 65 years) to achieve adequate representation in each category. In addition, the oldest group was defined according to the approximate retirement age.

The clinical implication of our study is that older patients with fibromyalgia appear to have a similar level of symptom severity as younger individuals. This may affect their functional ability and should be taken into account when planning treatment and rehabilitation for older patients. Our results also suggest that having a family member with fibromyalgia may be a predisposing factor for an earlier onset of fibromyalgia symptoms. However, further research with a larger study population is needed to clarify this association.

## Conclusions

In conclusion, there was no statistically significant association between patients with fibromyalgia who had a family history of the condition and their own symptom severity. The results provided no evidence of linear associations between age and symptom severity, nor between these outcomes and family history of fibromyalgia. Controlling for family history did not alter these conclusions. In our sample, patients with fibromyalgia across all age groups reported similar symptom severity and impact on functional ability, indicating that aging may not lead to symptom relief.

## Data Availability

The data that support the findings of this study are available from Nokia Health Center, but restrictions apply to their availability. The data were used under license for the current study and are therefore not publicly available. However, the data are available from the authors upon reasonable request and with permission from Wellbeing services county of Pirkanmaa.

## Abbreviations

ACR 2010: American College of Rheumatology’s 2010 diagnostic criteria for fibromyalgia
ANOVA: One-way analysis of variance
CWP: Chronic widespread pain
EQ-VAS: The EuroQol Visual Analogue Scale
FIQ: Fibromyalgia impact questionnaire
FM: Fibromyalgia
GP: General practitioner
hsCRP: high sensitivity C-reactive protein
ICD-10: International statistical classification of diseases and related health problems 10th revision
PSD: Polysymptomatic distress scale
SS: The symptom severity
WPI: The widespread pain index
